# Light-Responsive Polymer Micro- and Nano-Capsules

**DOI:** 10.3390/polym9010008

**Published:** 2016-12-29

**Authors:** Valentina Marturano, Pierfrancesco Cerruti, Marta Giamberini, Bartosz Tylkowski, Veronica Ambrogi

**Affiliations:** 1 Department of Chemical, Materials and Production Engineering (DICMAPI), University of Naples “Federico II”, P. le Tecchio, 80, 80125 Napoli, Italy; valentina.marturano@unina.it (V.M.); ambrogi@unina.it (V.A.); 2 Institute for Polymers, Composites and Biomaterials (IPCB-CNR), Via Campi Flegrei, 34, 80078 Pozzuoli (NA), Italy; 3 Department of Chemical Engineering (DEQ), Universitat Rovira i Virgili, Av. Països Catalans, 26, 43007 Tarragona, Spain; marta.giamberini@urv.cat; 4 Chemistry Technology Centre of Catalonia (CTQC), C/Marcel·lí Domingo, 43007 Tarragona, Spain; bartosz.tylkowski@urv.cat

**Keywords:** smart materials, photo-responsive capsules, interfacial polymerization, layer-by-layer, self-assembly, liposomes

## Abstract

A significant amount of academic and industrial research efforts are devoted to the encapsulation of active substances within micro- or nanocarriers. The ultimate goal of core–shell systems is the protection of the encapsulated substance from the environment, and its controlled and targeted release. This can be accomplished by employing “stimuli-responsive” materials as constituents of the capsule shell. Among a wide range of factors that induce the release of the core material, we focus herein on the light stimulus. In polymers, this feature can be achieved introducing a photo-sensitive segment, whose activation leads to either rupture or modification of the diffusive properties of the capsule shell, allowing the delivery of the encapsulated material. Micro- and nano-encapsulation techniques are constantly spreading towards wider application fields, and many different active molecules have been encapsulated, such as additives for food-packaging, pesticides, dyes, pharmaceutics, fragrances and flavors or cosmetics. Herein, a review on the latest and most challenging polymer-based micro- and nano-sized hollow carriers exhibiting a light-responsive release behavior is presented. A special focus is put on systems activated by wavelengths less harmful for living organisms (mainly in the ultraviolet, visible and infrared range), as well as on different preparation techniques, namely liposomes, self-assembly, layer-by-layer, and interfacial polymerization.

## 1. Introduction

In recent years, a growing interest has been focused on micro- and nano encapsulation due to their fruitful applications in controlled release of drugs [1], active agents [2], catalysts [3], and paints [4], as well as in synthetic nano-reactors engineering [5]. Academic and industrial research is particularly interested in so-called “environmentally responsive” materials, able to respond to an external stimulus (e.g., temperature, pH, light, electric or magnetic field) by modifying one or more of their intrinsic properties. For their adaptive features, these materials are often called smart [6,7]. One of the most challenging aspects of micro- and nano-encapsulation is the obtainment of a controlled and modulated release of the encapsulated—or core—material that can be achieved using smart materials as components of the capsule shell [8].

**Figure 1:**
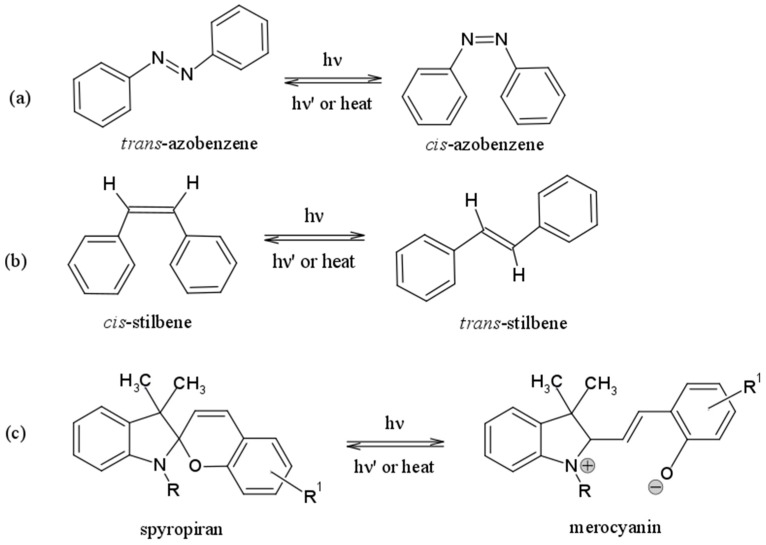
Photo-isomerization mechanism of photochromic molecules: (**a**) azobenzene; (**b**) stilbene; and (**c**) spiropyrane.

The design and development of high-sensitive systems, able to smartly recognize an external triggering factor and to respond by modifying their own structure, is the ultimate purpose of scientists all over the world. For this purpose, polymeric materials are particularly suitable for technical applications because they are versatile and their properties can be easily tailored depending on the final use. Many external stimuli, such as pH [9], temperature [10,11], biological molecules [12], and redox reactions [13] have been employed to effect capsule permeability or induce capsule disruption, enabling the release of the encapsulated material. Light (infrared, UV radiation or simply sunlight) is certainly the most compelling external stimulus, because it can be delivered without direct contact, thus representing one of the few remote-control triggering factors available [14]. Like many promising technologies, photo-responsive systems have been inspired by nature, which has evolved many complex biological systems able to exploit light as an external source of energy and information. For example, the light-induced *cis–trans* isomerization of the retinal molecule triggers a number of events, including a change in the conformation of the opsin protein to which is bound, leading to a neural signal and ultimately to the perception of light [15]. Mimicking natural structures, photo-responsive polymers can be obtained introducing photo-sensitive moieties in the polymeric backbone or in the side chains. Among the best performing photo-sensitive molecules, azobenzene [16], stilbene [17], and spiropyrans [18] stand out. The photoactivity of each of these functional groups is based on the existence of two interconvertible isomers. Upon light irradiation, typically in the ultraviolet range, the molecules undergo a conformational rearrangement. In the case of azobenzene and stilbene, this alteration is expressed by variations in the molecular symmetry from a thermally stable *trans* (*E*) orientation to a less favorable *cis* (*Z*) orientation (Figure 1a,b) [19]. In spiropyrans, the irradiation induces a ring-opening reaction that leads to the formation of the isomeric merocyanine form, as shown in Figure 1c [18].

One of the most interesting features of such photochromic materials is that isomerization is usually accompanied by molecular changes in physical properties such as polarity, viscosity and absorbance as well as macroscopic changes in material properties such as thickness, wettability and stability [20]. The presence of photo-responsive moieties in the capsule shell can therefore affect permeability of capsules or even lead to their disruption [21].

A key factor to take into account when designing photo-responsive micro- and nanocapsule systems is the wavelength of the light used to trigger the release. For outdoor use or other applications in which direct contact between light and capsules is granted, it is theoretically possible to employ any wavelength required by the photochromic materials that constitute the capsules shell. However, with regard to biomedical applications, the skin penetration depth of the light source involved in the release is the factor that determines the appropriate use of the capsules. The optical behavior of human skin upon light irradiation has been vastly studied and reviewed [22]. UV and visible light are reported having short penetration (few micrometers) depth and are most suitable for topical uses; on the contrary, near infrared light has a higher skin penetration depth of few millimeters and it could therefore be employed in internal delivery applications.

This review intends to give an overview on recent advances in the preparation of light-responsive polymeric capsules. Different preparation technologies will be discussed in detail, including interfacial methods (interfacial polymerization and phase inversion precipitation), template methods, and self-assembly methods. Capsules properties such as size, morphology and release behavior will also be described, with a view on the envisaged target applications.

## 2. Interfacial Methods for Capsules Formation

In interfacial methods, polymer capsules shell forms at the interface between two immiscible liquids. The first reaction at a liquid–liquid interface was performed in 1883 by Schotten and Baumann [23,24]. Since then, simple and versatile interfacial reactions, such as polycondensation, have been employed to overcome the challenging procedures in bulk or melt [25]. Interfacial polycondensation method is nowadays one of the most performing for in-situ formation of capsule shell [26]. Further, the interface between two immiscible liquids can also be used to precipitate a preformed polymer that will constitute the capsule shell [27]. In the following, the encapsulation at liquid–liquid interface with both polymerization and polymer precipitation will be discussed.

### 2.1. Emulsion Polymerization

Interfacial polymerization has been widely described in literature, and used for the realization of thin films [28] and particles [29]. This technique can also be employed for the preparation of micro- and nanocapsules [30,31] when supported by an emulsification step. An emulsion is defined as a dispersed system of liquid droplets (dispersed phase) in another, non-miscible liquid (continuous phase), stabilized by means of one or more surfactant agents. In the preparation of core–shell structures, the most performing interfacial reactions are polycondensation and polyaddition due to their simple mechanism, fast kinetics and high yields [26,32]. The polycondensation reaction occurs between different multifunctional monomers, either dissolved in the droplet suspension or in the continuous phase. The monomers react at the interface of the emulsion droplets forming the primary membrane, and the polymerization reaction advances until the depletion of one of the monomers. The typical hollow structure is obtained when the formed polymer is not soluble in the core material [31]. The common approach to obtain a photo-responsive shell membrane is employing photochromic monomers in the polycondensation reaction. For example, Tylkowski et al. [33] proposed a new approach for the preparation of liquid crystalline polyamide microcapsules containing azobenzene mesogens in the main chain. The triggered release of the encapsulated β-carotene was successfully performed by irradiating the capsules with 365 nm UV-light. At this wavelength, *trans–cis* photo-isomerization of azobenzene occurs, leading to major rearrangements in the macromolecules conformation that eventually result in the release of the encapsulated material.

**Figure 2:**
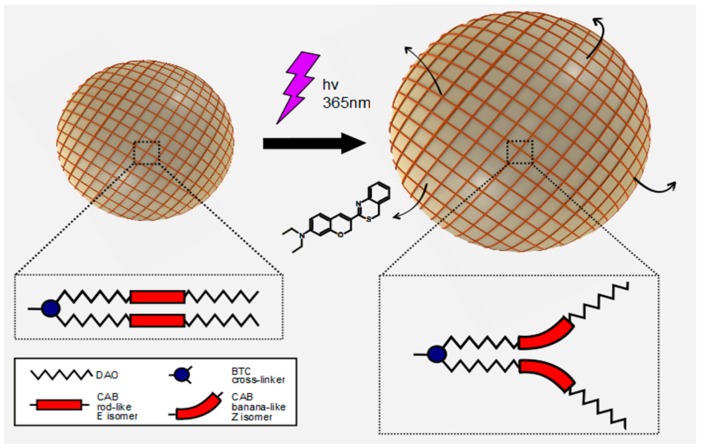
Schematization of the C6 release from photo-responsive polymer nanocapsules as depicted by Marturano et al. [34]. Reproduced with permission from Elsevier.

In order to scale the dimensions of the capsules down to the nanometer range, the polycondensation reaction described above has been combined with a miniemulsification step. Miniemulsions are a special class of emulsions, produced via high-energy homogenization (e.g., high shear stirring or ultrasonication), stabilized against coalescence and molecular diffusion degradation, and characterized by a narrow droplet distribution [32]. Marturano et al. [34] successfully reported the preparation of photo-responsive polyamide nano-sized capsules. The authors described how simple miniemulsion parameters, such as surfactant type and concentration affect key final properties, such as capsules dimension and release behavior. Release experiments of fluorescent probe molecule coumarin-6 (C6) confirmed the successful light-triggered release. Interestingly, dynamic light scattering (DLS) measurements demonstrated that the average diameter of the capsules significantly increased on UV exposure due to the rearrangement of the polyamide shell from a “closed” to a more “open” conformation, as depicted in Figure 2.

Micro- and nanocapsules described above can meet the target of many specific applications, depending on their size and release profile, and serve as carriers for the encapsulation and release of different active agents. For example, Bizzarro et al. [35] reported the successful encapsulation and release of cumin and basil essential oils.

One of the great advantages of the described systems is the formation of robust capsules. The release of the core material occurs by leakage as a consequence of changes in shell permeability, without compromising shell integrity. This mechanism makes capsules safer for biological and medical applications, differently from systems where fragments derived from shell disruption can possibly contaminate target environment. On the other hand, one of the main drawbacks is the use of UV light, since this wavelength range has limited use in biological in vivo applications [36] and its concentration in sunlight is too scarce to be employed in agricultural or packaging applications. Beharry et al. [37] and Wegner [38] demonstrated how the incorporation of electron-donating groups in *ortho* or *para* position on the azo moiety can dramatically red-shift the photoswitching wavelength. Taking advantage of this work, Tylkowski et al. [39] were able to synthesize modified polyamide microcapsules shell containing *ortho*-substituted azobenzene moieties. It was shown that this modification led to an increase in shell permeability and release of core material under visible light irradiation.

A new frontier in the preparation of polymeric capsules is the use of microfluidic systems in which low volumes of fluids are processed through automatic and high-yield mechanism to obtain narrowly distributed droplets [40]. For example, interfacial polymerization reactions have been successfully performed in microfluidic devices [41]. Recently, Zeng et al. [42] reported the self-assembly of photo-responsive reversibly cross-linked hydrophilic and hydrophobic copolymers that can be controllably brought together at the water–chloroform interface of a microfluidic droplet. The cross-linking agent consists in a ternary host–guest complex containing azobenzene, whose UV-triggered *trans–cis* isomerization leads to the reversible disruption of the supramolecular assembly and consequent release of the cargo material.

An alternative approach to obtain photo-responsive microcapsules is employing metal or metal oxide nanoparticles acting as light absorbers. Chen et al. [43] obtained polystyrene microcapsules via Pickering emulsion polymerization using modified SiO_2_ and TiO_2_ nanoparticles as Pickering agents. The release of the encapsulated material was achieved by degradation of the polymeric shell caused by the photocatalytic activity of TiO_2_ nanoparticles [44].

### 2.2. Phase Inversion Precipitation

As mentioned before, an alternative approach to the synthesis of polymeric carriers is the use of preformed polymers for the capsules shell. Bogdanowicz et al. [45] successfully employed a novel photo-responsive polymer, containing photochromic stilbene moieties in the main chain, poly(α-methylstilbenesebacoate-*co*-α-methylstilbeneisophthalate) (P4), as shell material for vanillin loaded microcapsules. The capsules preparation was based on phase-inversion precipitation procedure, previously optimized by Peña et al. [46]. Using a nozzle device connected to compressed air flow, a homogeneous polymer solution was broken into microdroplets and sprayed in a coagulation bath containing a non-solvent. Precipitation of the polymer at the interface of each droplet was caused by exchange of solvent and non-solvent molecules in contact with the polymer. The authors hypothesized that the overall change in the shell permeability may be due to cooperative rearrangements of the polymeric chains induced by the photo-isomerization of the photo-responsive α-methylstilbene.

## 3. Templating Methods

This section intends to include different examples of micro- and nanocapsules formed via deposition of polymer material on colloidal sacrificial particles serving as template for the formation of hollow structures. The most acknowledged templating method is the layer-by-layer (LbL) approach, based on the consecutive deposition of interacting polymers on a sacrificial template particle which can be removed at the end of the process [47].

### 3.1. Layer-by-Layer (LbL) Using Polyelectrolytes

**Figure 3:**
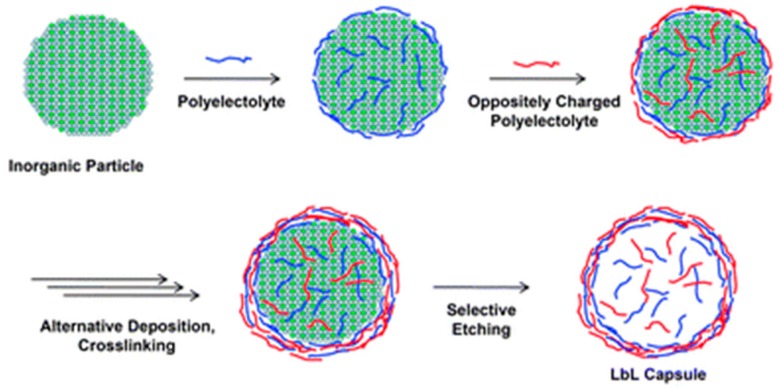
Formation of polyelectrolyte based layer-by-layer nanocapsule as schematized by Yoon et al. [50]. Reprinted with permission from [50]. Copyright 2010 Royal Society of Chemistry.

A wide variety of LbL capsules can be found in literature [48,49], however the vast majority of LbL capsules has been prepared using polyelectrolytes. The procedure, schematized in Figure 3, involves alternating deposition of positively and negatively charged polyelectrolytes onto the template, where the driving force for the assembly is the electrostatic interaction. After deposition, polymers can be cross-linked and, finally, hollow capsules are obtained by selective etching of the inorganic template [50].

A wide range of materials, both synthetic and bio-based, are suitable candidates to form the shell, and the range of particle sizes spans from the nanometer to several micrometers, mostly depending on the size of the template. The main challenges concerning the preparation of nano-sized LbL capsules are related to aggregation phenomena. However, this size range cannot be neglected since is particularly important for in vivo applications. On the other hand, micro-sized capsules are very attractive objects because of the simplicity of their characterization and imaging, facile prevention of aggregation and superior loading capacity [51]. The surface of the capsules has been frequently modified in order to tailor the capsules properties to the final application requirements, such as improved colloidal stability, enhanced confinement of the encapsulated core substances or incorporation in the polymer shell of active materials for imaging and sensing [52].

Tao et al. [53] published in 2004 an early example of a LbL capsule system containing an azo dye in the shell. Negatively charged Congo red (CR), bearing two negative charges and a chromophore moiety, was deposited on a melamine-formaldehyde sacrificial template alternated with positively charged polyelectrolyte. The presence of CR in the capsules shell imprinted brand new properties to the polymer capsules. In particular, the permeability of the shells could be remotely controlled irradiating the capsules with visible light. A similar example of photo-responsive LbL capsules, based on azobenzene moieties, was proposed by Bédard et al. [54]. In this case, the LbL procedure involved alternate absorption of sodium salt of azobenzene, poly(vinylsulfonate) and poly(allyamine hydrochloride) layers. The permeability changes were caused by the *trans–cis* photo-isomerization of azobenzene. Experimental results showed that exposure of microcapsules to light led to significant shrinking, increased roughness and enhanced permeability of the capsule shell. Moreover, the authors reported the successful encapsulation of a fluorescent probe macromolecule and its release upon light irradiation.

**Figure 4:**
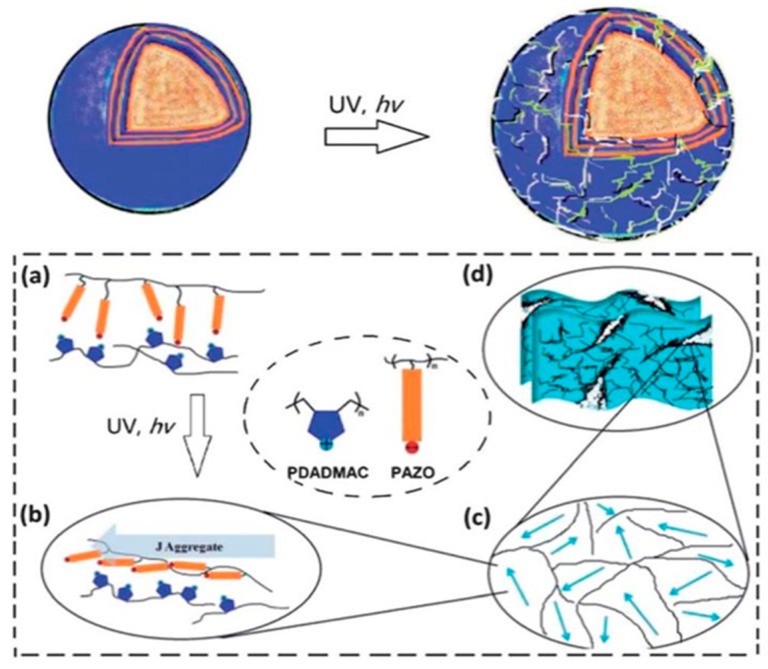
Schematic illustration of (PDADMAC/PAZO) microcapsule disruption induced by UV irradiation [55]: (**a**) LbL assembly of the polyelectrolytes on the capsule shell surface; (**b**) formation of J aggregates under UV irradiation; (**c**,**d**) extended aggregates act as stress raisers, triggering capsule breakage. Reprinted with permission from [55]. Copyright 2014 Royal Society of Chemistry.

In 2014, Yi and Sukhorukov [55] reported on LbL UV-responsive microcapsules made of alternating layers of negatively charged poly[1-[4-(3-carboxy-4-hydroxyphenylazo)benzenesulfonamido]-1,2-ethanediyl sodium salt (PAZO) and poly(diallyldimethyl ammonium chloride (PDADMAC). In this case, the photo-responsive behavior was attributed to the presence of PAZO segments, that upon UV-light irradiation rearrange forming J aggregates. The schematic illustration of PDADMAC/PAZO microcapsule disruption is reported in Figure 4. Extensively investigated in the literature [56], J aggregates are small aggregates, constituted by three or four monomeric units having the same orientation and created via strong non-covalent aromatic-aromatic interaction. These formations are not flexible enough to retain the spherical structure of the shell, so that the capsule gradually breaks, swelling and leaking the core material, until final disruption.

**Figure 5:**
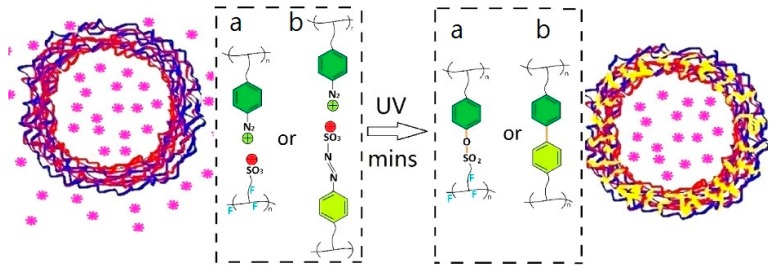
Photolysis-induced small molecule encapsulation in: (**a**) Nafion/DAR; and (**b**) DAR single component multilayer capsules as depicted in [57]. Reprinted with permission from [57]. Copyright 2013 American Chemical Society.

Release experiments were performed on the capsules loaded with a model core substance, bovine serum albumin (BSA). The results showed how the capsules disruption process could be modulated to control the release of the encapsulated BSA by adjusting the UV intensity and microcapsule architecture. However, it was noticed that BSA molecules were able to leak through the porous multilayer shell even without the support of UV-light. To overcome this problem, the same authors developed a very interesting multifunctional capsule system in which UV response was time-dependent and involved both encapsulation and release processes [57]. This approach was specifically designed to promote the confinement of low molecular weight water-soluble substances that usually are very prone to leak from the capsule due to its intrinsic porosity. Instead of increasing the density of the multilayer to obtain a decrease of permeability, Yi and Sukhorukov proposed a chemical sealing of diazoresin (DAR)-containing microcapsules. In both Nafion/DAR and DAR single component [58] microcapsules, irradiation with UV light at 380 nm led to photolysis of the interacting ion pairs, causing the decomposition of the diazonium group, and the formation of a sulfonate covalent bond, as shown in Figure 5. The photo-induced conversion from ionic to covalent chemical bonds via DAR photolysis offers an externally controlled method to seal the multilayer capsules and guarantee minimal diffusion of the encapsulated molecules. Interestingly, UV-sealed capsules showed a more efficient preservation of Rhodamine B, over storage time, than their un-irradiated counterparts.

The great advantage of this method is that more interesting low molecular weight substances could be encapsulated in the DAR capsules without changing environmental conditions, such as ionic charge [59] or pH [60]. Moreover, the UV-induced rapid capsule sealing would be extraordinarily useful in terms of catching and analyzing small molecules in a biological environment.

### 3.2. Layer-by-Layer (LbL) Using Host-Guest Systems

**Figure 6:**
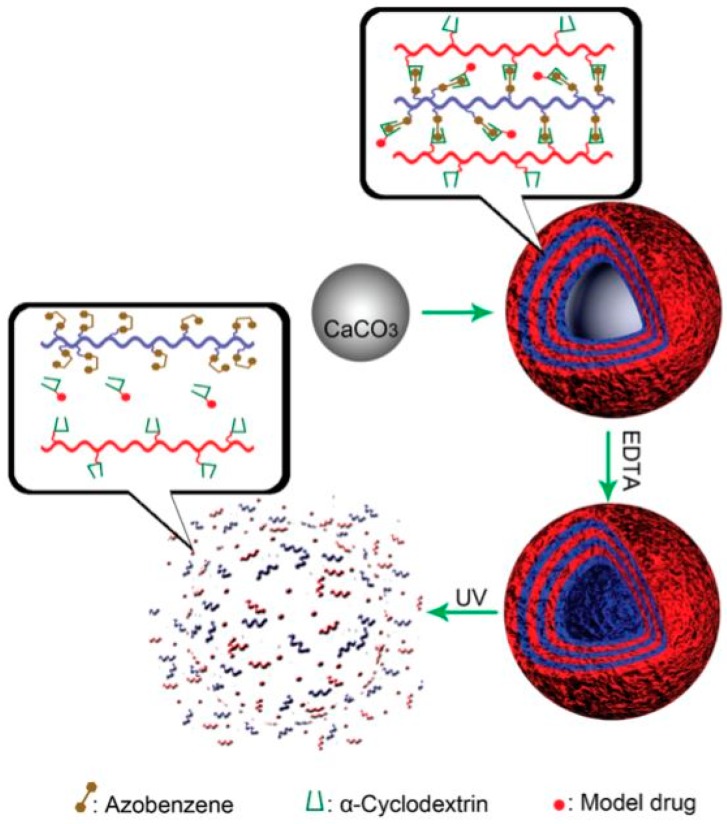
Capsules structure and release mechanism of α-CD/Azo LbL microcapsules as depicted by Xiao et al. [62]. Reprinted with permission from [62]. Copyright 2011 American Chemical Society.

For a long time, the only driving force of the LbL technique has been the electrostatic interaction between polyelectrolyte pairs, therefore the limited amount of oppositely charged and water soluble polymers available for the process constituted the main drawback of this technique. A possible alternative to electrostatic-driven LbL structures are supramolecular assemblies, a set of molecules held together by non-covalent bonds. These structures can be formed by just two molecules (e.g., DNA double helix) or, more often, by a great amount of molecules able to form complex structures such as spheres, rods or sheets (e.g., micelles, liposomes and biological membranes). In the domains of supramolecular chemistry, the development of host–guest systems, in which a host molecule can recognize and bind a certain guest molecule, was considered as an important contribution. A host–guest system refers to a chemical system that is made up of two or more molecular subunits self-assembled together to form a supramolecular complex. Normally, the formation of a host–guest system involves more than one type of noncovalent interaction, for example, hydrophobic association, hydrogen bonding, electrostatic interactions, metal coordination, van der Waals forces, and π–π stacking interactions [61]. In this frame, Xiao et al. [62] successfully obtained photo switchable microcapsules based on host–guest interaction, using a host layer containing α-cyclodextrin (α-CD) and a guest layer based on azobenzene (Azo) assembled on sacrificial CaCO_3_ particles via LbL deposition. α-CD-rhodamine B (α-CD-RhB), used as a model drug, was loaded on Azo layers by host–guest interaction. Interestingly, under UV irradiation (λ = 365 nm) a modification of the host guest interaction occurred, mainly due to Azo isomerization, leading to the disruption of the capsules shell and the release of the encapsulated drug. The capsules structure and the release mechanism are depicted in Figure 6. The release of the modified α-CD was successfully monitored through spectrofluorometric analysis thanks to the modification of the model drug with the fluorescent rhodamine B. The experiment showed how the drug release from the sample irradiated with UV light was dramatically faster compared to the un-irradiated sample.

**Figure 7:**
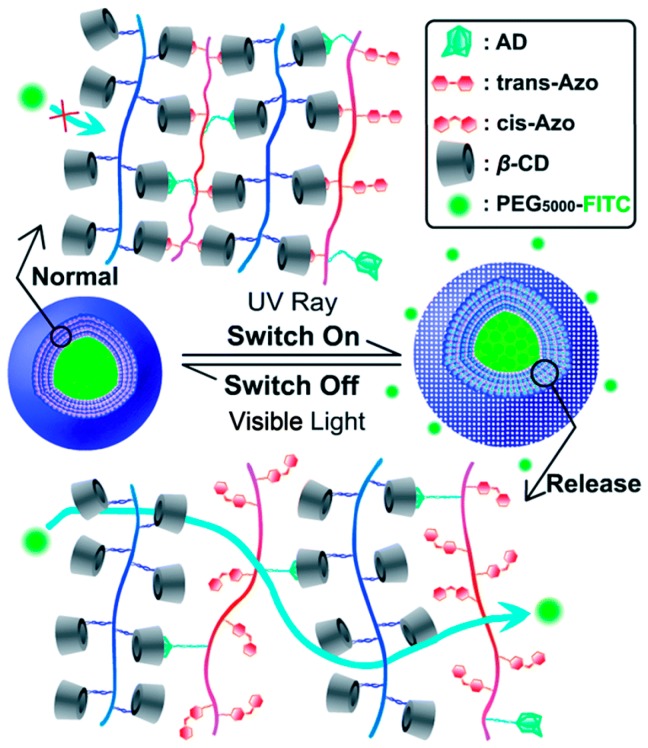
On/off photo-responsive switch in the LbL microcapsules designed by Lin et al. [63]. Reprinted with permission from [63]. Copyright 2014 Royal Society of Chemistry.

A further implementation of the supramolecular LbL approach was provided by Lin et al. [63]. The LbL assembly was driven by two different host–guest interactions, one between adamantine (AD) and β-cyclodextrin (β-CD) and one between azobenzene (Azo) and β-CD. The versatility of β-CD allows it to accept both AD or Azo as a guest molecule into the inner hydrophobic chamber [64,65]. In particular, the *trans*-Azo isomer is suitable for entering the inner chamber of β-CD while the *cis*-Azo isomer shows no supramolecular interaction because of steric hindrance. As a result, UV photo-irradiation could cause the dissociation of β-CD/Azo complex. The microcapsules designed by Lin et al. are able to controllably switch between the “on” and “off” state. As shown in Figure 7, the stable host–guest interaction between β-CD and AD maintains the structural integrity of the shell, while the reversible UV-sensitive interactions between Azo and β-CD could form a dense membrane to confine the drug. Under UV light irradiation (λ = 365 nm) the photo-switching of Azo from *trans* to *cis* implies a weakening of the Azo/β-CD interactions and a decrease in the density of the layers, and the consequent diffusive release of the encapsulated molecule. Release experiments of the encapsulated fluorescent PEG_5000_-FITC probe drug confirmed the reversible switching between “on” and “off” state as a proof of concept of the “release-cease-recommence” mechanism.

### 3.3. Other Templating Methods

It is worth mentioning another example of LbL capsules based on photo-responsive moieties different from azobenzene. Achilleos et al. [66] engineered LbL nanocapsules based on photosensitive spiropyrans (SPs) moieties. Upon UV irradiation, non-polar SPs isomerize to merocyanines (MCs); the process is reversible, since under visible-light irradiation MC regenerates the SP form [67]. The supramolecular design of these capsules was based on the intrinsic feature of MCs to aggregate into either H- or J-type stack-like arrangements through noncovalent π–π interactions.

In the class of templating methods, LbL is by far the most technologically advanced. However, other methods for the formation of hollow capsules based on a sacrificial particle as template can be found in literature. For example host–guest interactions between cyclodextrin-appended polymers (host) and complementary ferrocene or azobenzene carriers (guest) was employed by Wajs et al. to obtain stimuli-responsive nanocapsules using sacrificial golden colloidal templates [68]. Li et al. [69] introduced a facile method to fabricate photo-responsive capsules, using a *ortho*-nitrobenzyl derivative as cross-linking agent for polyethyleneimine (PEI) and CaCO_3_ templating particles. The release of the encapsulated model cargo under UV light irradiation occurs because of the photo-cleavable nature of the cross-linking points [70,71], leading to capsules dissociation.

For biomedical applications that involve laser-nanoparticle interaction, the light needs to guarantee both minimum absorption by cells/tissue and maximum absorption by nanoparticles. The ideal light source is the so-called biologically “friendly” wavelength window [72]—the near-infrared (NIR) part of the spectrum. Light-responsive capsules have the potential for in vivo drug delivery because NIR light is much less harmful and has a much deeper penetration depth in tissues compared with UV or visible light. However, photo-responsive polymer moieties that typically constitute the polymeric capsules shell are inert to IR light, so functionalization of the capsules shell with noble metal nanoparticles becomes necessary [73]. These particles are able to efficiently absorb laser energy and convert it into heat, which locally and transiently dissipates to a polyelectrolyte network. For example, Skirtach et al. proposed polyelectrolyte-multilayer microcapsules carrying silver nanoparticles embedded in their shell. It was possible to remotely activate the capsule, injected in living cells, by irradiation with near-IR light [74,75].

**Figure 8:**
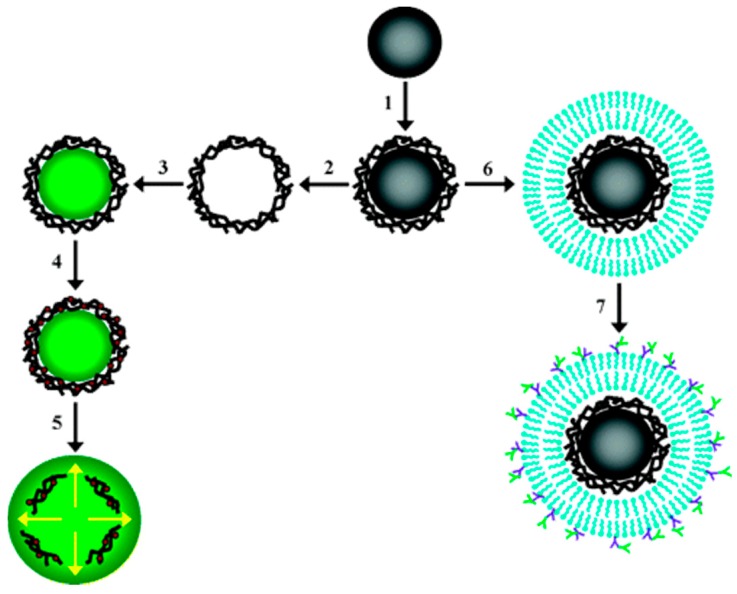
Schematic illustration of the various colloidal systems investigated by Angelatos et al. [76]. Reprinted with permission from [76]. Copyright 2005 American Chemical Society.

Similarly, Angelatos et al. [76] reported the preparation of NIR-responsive capsules prepared via LbL-assembly of polyelectrolytes using melamine formaldehyde particles as sacrificial templates. Exploiting the pH-dependence of the shell permeability, modified dextran was succesfully loaded into preformed capsules. Subsequently, infiltration of light-absorbing gold nanoparticles into the capsule shell was performed to render the capsules optically addressable. A schematization of the process is reported in Figure 8.

The authors demonstrated that is possible to tune the release of the encapsulated material irradiating the capsule with a short-pulse (10 ns) NIR laser light (λ = 1064 nm). Moreover, the polyelectrolyte shell was coated with a lipid bilayer, increasing capsules bio-recognition capabilities [77].

Such capsules are likely to have potential as delivery vehicles for drug administration, microreactor applications, and even cell manipulation. Ambrosone et al. [78] reported an interesting application of NIR-responsive LbL capsules for advanced in vivo delivery of an intracellular modulator of Wnt/β-catenin signaling pathway. The relevance of this work lays in the importance of controlling cell function and reprogramming cell fate upon external triggering.

## 4. Self-Assembly Methods

The spontaneous formation of non-covalent association of organic molecules in solution is commonly called self-assembly. Scientists are very intrigued by this phenomenon, mainly because of the intrinsic compelling nature of self-ordered structures, but also because this structures naturally occur in living organisms [79]. The formation of hollow carriers is often enabled by the use of amphiphilic molecules, characterized by both hydrophilic and hydrophobic parts. In the following section, different preparation methods of self-assembled micro- and nanocapsules, based on amphiphilic block copolymers and low molecular weight amphiphiles are reported.

### 4.1. Block Copolymers Self-Assembly

The formation of micelles from self-assembly of block copolymers in a selective solvent has been known since 1970s [80]. Recently, self-assembled polymer capsules have been used to encapsulate drugs and other active agents as well as enzymes and non-biologic catalysts, serving as nanoreactors [81]. Different approaches have been developed to obtain targeted drug delivery via tuning the amphiphilicity of the block copolymers. In particular, Blasco et al. [82] reported a new family of photo-responsive self-assembly formulations based on a series of amphiphilic linear–dendritic block copolymers (LDBCs) containing photochromic azobenzene units and hydrocarbon chains randomly connected to the periphery of the dendron. One of the main drawbacks of this technique is the use of organic solvents and complicated preparation procedures of the block copolymer units. The same authors proposed a simpler synthetic approach compared to the former design, based on an azobenzene-containing miktoarm polymer that formed stable vesicles, able to load and release both hydrophobic and hydrophilic cargo molecules upon UV irradiation [83].

**Figure 9:**
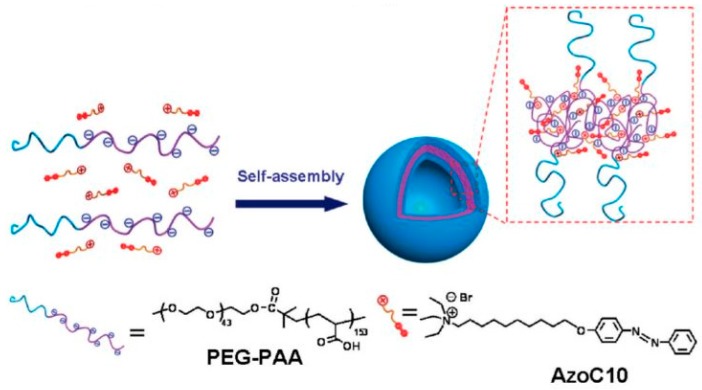
Schematic illustration of the self-assembly of block ionomer complex vesicles as depicted by Wang et al. [86]. Reprinted with permission from [86]. Copyright 2009 American Chemical Society.

To overcome the problems related to the use of organic solvents, efforts have been done in the development of block copolymer assemblies based on electrostatic interactions [84,85]. Water is a suitable solvent for this novel class of polymeric assemblies, since they are formed by double-hydrophilic block copolymers, containing ionic and nonionic water-soluble segments (block ionomers). A new frontier in ionomer self-assembly was reported by Wang et al. [86]. They introduced stimuli-responsive moieties onto surfactant molecules, so that surfactant aggregates can be tuned toward controllable disassembly. The UV-induced variation of the critical micellar concentration (CMC) of the *trans* and *cis* forms of azobenzene-bearing surfactants is a well-known process, that can be used to induce the destruction/formation of micellar structures. The strategy employed to prepare vesicles based on block ionomer complex is reported in Figure 9. UV-vis spectrophotometry tests demonstrated that the molecules of azobenzene-containing surfactant included in the block ionomer complex were able to undergo *trans*-to-*cis* isomerization if irradiated with UV light at 365 nm, and reversibly switch back from *cis* to *trans* form if irradiated with visible light at 450 nm.

### 4.2. Liposomes

**Figure 10:**
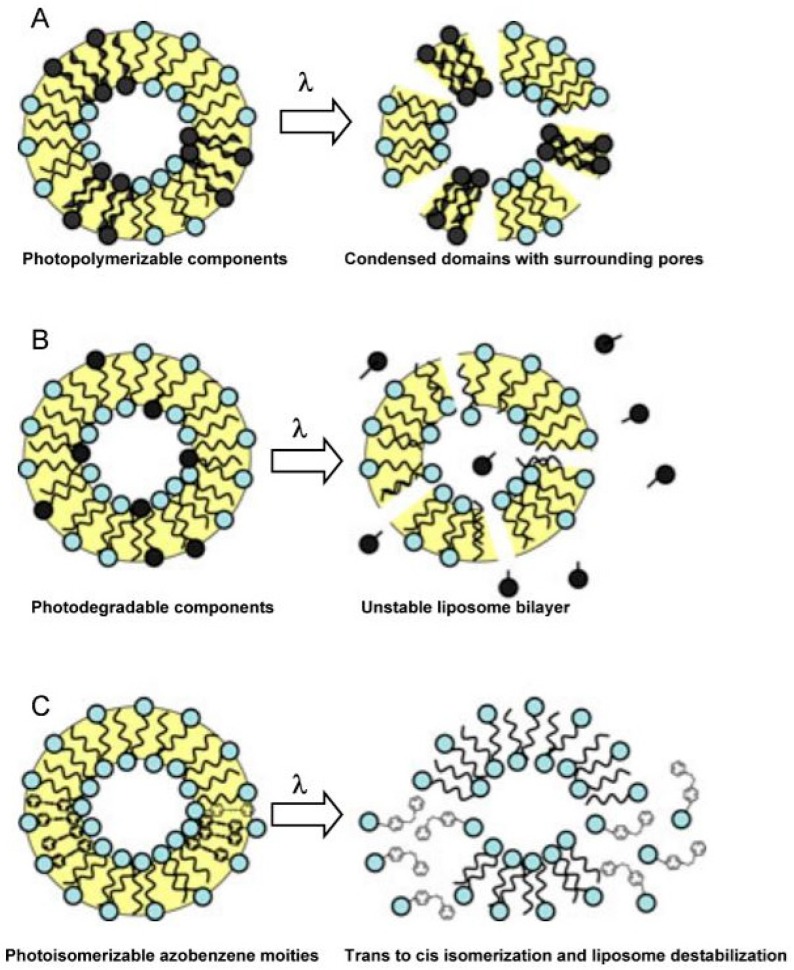
Schematization of light-triggered release mechanisms in liposomes by inclusion of: (**A**) photo-polymerizable components; (**B**) photodegradable components; or (**C**) photo-isomerizable azobenzene moieties. Reprinted with permission from [92]. Copyright 2012 Ivyspring International Publisher.

Liposomes consist of concentric bilayers of phospholipids and/or other amphiphilic molecules encapsulating an aqueous compartment, resulting in nanosized vesicles. Intensive studies have been carried out on the encapsulation of drugs in liposomes, as they are promising carriers in aqueous fluids [87,88]. Among other drug carriers for cancer treatment, liposomes are the longest-studied nanoparticles and are hence associated with a number of historic milestones [89] Despite improvements in the therapeutic efficacy versus side effects obtained in the dosage of few relevant drugs (e.g., amphotericin B and doxorubicin), the desired drug release from liposomes is still a challenge [90]. One of the main drawbacks of liposome carriers is the passive release by diffusion of the encapsulated drug. In most cases, diffusion occurs too slowly and the local drug concentrations required for the optimum therapeutic effect are not reached [91]. Rapid and targeted drug delivery can be achieved triggering chemical and physical changes in liposome shell using external light irradiation [92], as illustrated in Figure 10.

The mechanism depicted in Figure 10A is based on photo-polymerization of membrane lipids. The application of a proper light source induces photo-polymerization of reactive molecules (bearing dienoyl, sorbyl or styryl groups) introduced into the liposome membrane. This leads to the formation of condensed domains in the bilayer; at the same time pores are temporarily formed around the clusters until the surrounding free mobile lipids rearrange to reconstitute the bilayer. Such pores allow drug molecules to diffuse out of the liposome. For example, Bondurant et al. [93] showed that the inclusion of a photo-reactive lipid component in PEG-liposomes membrane did not alter the permeability of liposomes prior to irradiation, while exposure to UV light (λ = 254 nm) for 2 min led to an increased liposome permeability.

**Figure 11:**
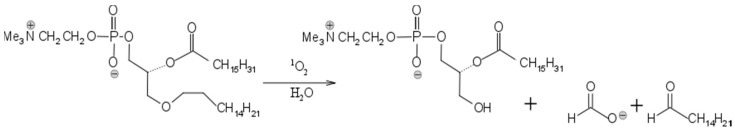
Singlet oxygen-mediated photo-oxidation of plasmalogen vinyl ether linkage.

Light-responsiveness of liposomes can also be photo-chemically triggered applying various chemical stimuli responsible for the destabilization or disruption of specific components of the liposome membrane (Figure 10B). One of the earliest examples was provided by Thompson et al. [94]. Their approach was based on the photo-cleavage of plasmenylcholine to single chain surfactants via sensitized photooxidation of the plasmalogen vinyl ether linkage (Figure 11). The authors presented the photo-triggered behavior of plasmenyicholine liposomes containing three different sensitizers absorbing between 630 and 820 nm.

In this frame, Luo et al. [95] demonstrated that the introduction of a small amount of an unsaturated phospholipid accelerates NIR light-triggered doxorubicin release in porphyrin–phospholipid (PoP) liposomes. The mechanism of the enhanced release rate was related to the oxidation of unsaturated phospholipids by singlet oxygen. In vivo studies demonstrated the efficiency of these systems in chemo-photo-therapy. Sine et al. [96] reported in vivo release studies of a novel photo-cleavable liposome system with projected applications for cancer treatment. In this case, the inclusion of a red absorbing photosensitizing agent in the liposome membrane was able to induce destabilization of “pockets” structures, resulting in defects in the liposome bilayer and causing the release of encapsulated drug.

**Figure 12:**
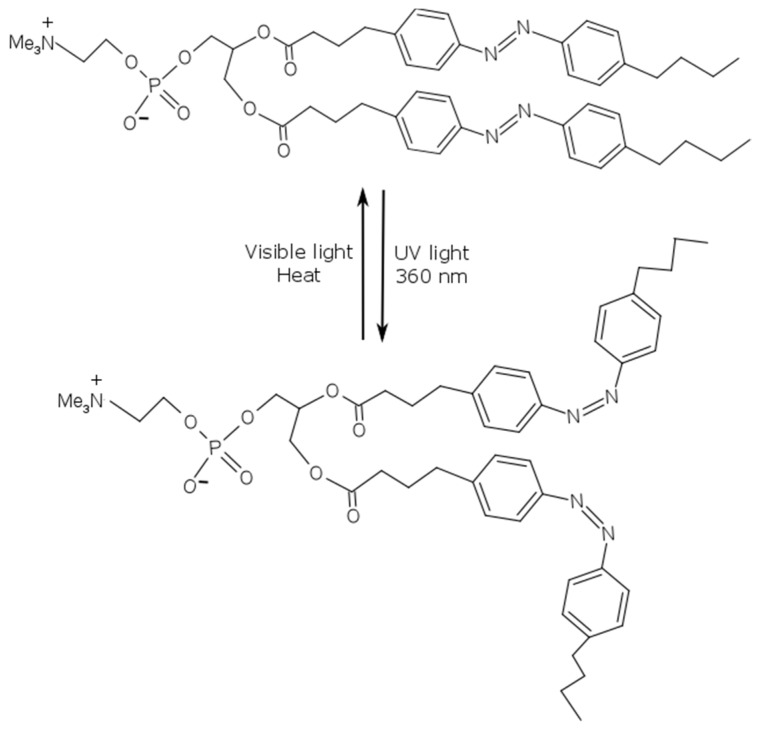
Mechanism of *trans–cis* isomerization under UV light irradiation of the photochromic lipid, Bis-Azo PC.

One of the most common approaches for the formation of light-responsive carriers is the introduction of photo-isomerizable lipids in the liposome membrane, as schematized in Figure 10C. Azobenzene-modified lipids (Bis-Azo PC) can undergo photo-isomerization, leading to photo-induced conformational changes in the liposomes. The *trans* to *cis* isomerization of the azobenzene groups alters the polarity and conformation of the lipids in a rapid and reversible process, as reported in Figure 12. This approach can guarantee one of the finest control of drug release by simply adjusting the liposome composition. For example, Bisby et al. [97] reported that an increase in cholesterol content enables to lower the photo-isomerization extent necessary to trigger the release, increasing the light sensitivity of azobenzene-containing liposomes.

More recently, Cui et al. [98] demonstrated the feasibility of fluid-phase photo-sensitive liposomes not based on phospholipids that combine very low passive permeability and good photo-control of the entrapped payload. The presence of the azobenzene derivative makes these liposomes sensitive to light and allows high-precision control on the release of the encapsulated material. The authors pointed out that the *trans* form of the azobenzene was compatible with the molecular packing of the bilayer, giving impermeable membranes. On the other hand, the *cis* form introduced defects in the tightly packed alkyl chains of the bilayer, allowing the photo-induced leakage of the encapsulated material.

Interesting advances in NIR-responsive liposomes consist in a new family of water-in-oil-in-wall (W/O/W) core–shell nanocapsules made from the self-assembly of proteins in a liposome-like double layer intercalated with reduced graphene oxide (rGO) nanosheets [99]. The rGO nanosheets are introduced to minimize unintended drug leakage, but it also serves as the NIR sensor/actuator that triggers drug release.

In a frontier application, multilayer capsule solely based on graphene oxide were tested as controlled drug delivery carriers [100], opening a novel way for NIR-light triggered release in a simple way without addition of nanoparticles or dyes.

## 5. Characterization Methods of Photo-Responsive Capsules

It is worth providing a short outlook on the most used [101,102,103] methods for collecting valuable data to characterize photo-responsive polymers. In Table 1 the classification of characterization techniques is based on different key capsules properties, namely: shape, size distribution, cross section and surface morphology, surface chemical analysis, thermodynamic properties of shell and encapsulated material, and release and stability of encapsulated material.

## 6. Conclusions

Significant progress in the design and the synthesis of light-responsive polymer micro- and nanocapsules has been made in recent years. Diversification of capsule preparation techniques and fine-tuning of materials chemical design provide an almost infinite number of strategies to obtain a customer-tailored application. However, many challenges need to be addressed, concerning both academic research and industrial application. Understanding the principles of the mechanisms at the basis of these stimuli-responsive materials is essential for developing novel encapsulation, release, and targeting methods.

The ultimate challenge for light-triggered delivery of drugs or other active agents in biological environments is to grant the use of biocompatible materials and un-harmful release process in use. Among the wide variety of photosensitive capsules available, a sensitive factor is the choice of an appropriate size range of delivery systems. Microcapsules, for example, have been widely studied and exploited in commercial applications for their facile preparation and characterization. On the other hand, biological application, such as circulation or cellular uptake experiments, have desperate need of nanocapsules.

Research and development in nano-sized range is currently experiencing a burst development and is in constant need for new carriers to further impact theranostics, nanomedicine and drug delivery.

## Figures and Tables

**Table 1:** Characterization techniques of photo-responsive capsules.

Capsules properties	Method
Capsule shape and size	Optical microscopy
	Dynamic Light Scattering (DLS)
	Particle size analyzer
Capsule shape, size and surface/cross-section morphology	Environmental/Scanning Electron Microscopy (ESEM/SEM)
	Transmission Electron Microscopy (TEM)
Capsule surface physical properties	Atomic force microscopy (AFM)
	Contact angle measurement (CA)
	Nanoindentation
Capsule surface chemical properties	SEM + X-ray microanalysis (EDS)
	X-ray photoelectron spectroscopy (XPS)
	Nuclear magnetic resonance spectroscopy (NMR)
	Attenuated total reflectance infrared spectroscopy (ATR-IR)
Thermodynamic properties of shell and/or encapsulated materials	Differential scanning calorimetry (DSC)
	Thermogravimetry (TG)
Active material stability and release	Ultraviolet-visible spectrophotometry (UV–Vis)
	Gas chromatography–mass spectrometry (GC–MS)
	High-performance liquid chromatography (HPLC)
	Spectrofluorimetry
	Olfactive Evaluation
